# Effects of Melatonin Administration on Physical Performance and Biochemical Responses Following Exhaustive Treadmill Exercise

**DOI:** 10.3390/cimb46120815

**Published:** 2024-11-30

**Authors:** César Berzosa, Pablo Jesús Bascuas, Eduardo Piedrafita

**Affiliations:** Faculty of Health Sciences, Universidad San Jorge, Autov. A-23 Zaragoza–Huesca Km. 299, 50830 Villanueva de Gállego, Zaragoza, Spain; pbascuas@usj.es (P.J.B.); epiedrafita@usj.es (E.P.)

**Keywords:** tissue biomarkers, lipid peroxidation, protein oxidation, membrane fluidity, ergogenic, brain, muscle, liver

## Abstract

Exercise, despite being a beneficial activity for health, can also be a source of oxidative imbalance, which can lead to a decrease in performance. Furthermore, melatonin is an endogenous molecule that may counteract exercise-induced oxidative stress. The aim of this study was to evaluate the potential ergogenic and antioxidant capacity of melatonin administered for a maximal effort test. A total of 30 rats were divided into three groups—control, exercise, and exercise + melatonin (intraperitoneal administration of 10 mg/kg)—to assess the effects of an exhaustive incremental protocol in the two exercise groups (with and without melatonin) on the treadmill-running performance (final speed reached), lipid and protein oxidation markers (malondialdehyde + 4-hidroxyalkenals and carbonyl content, respectively), and cellular and mitochondrial membranes’ fluidity in skeletal muscle, brain, and liver tissues. Our results show an ergogenic effect of melatonin (31 ± 4 vs. 36 ± 4 cm/s), which may be due to its antioxidant properties being significantly stronger than its protective effect when performing increasing exercise on a treadmill until exhaustion. Melatonin reverted the membrane rigidity in the brain caused by exercise (with no effect on muscle or liver), prevented lipid oxidation in muscle, and prevented lipid and protein oxidation in the liver. Differences between tissues’ responses to exercise and melatonin need to be investigated in the future to elucidate other possible mechanisms that explain melatonin’s ergogenic effect.

## 1. Introduction

Since the 1970s, it has been suggested that exercise is a cause of oxidative stress, considering that the muscle mitochondria are the principal source of free radicals during exercise [1,2]. However, at this moment, a lot of different sources of reactive species, including sarcolemma enzymes, such as xanthine oxidase [3], phospholipase A [4], or nitric oxide synthase, have been identified [5]. It is suggested that the changes in redox balance during exercise are promoted by non-muscle sources such as phagocytes. Infiltrated macrophages, essential to muscle repair, may release a huge amount of reactive species, leading to oxidative damage even in healthy muscle [6,7]. As a result, oxidative stress damage leads to early fatigue, diminishing exercise performance.

On the other hand, these free radicals have an important role as signals for adaptation responses when they are present in low concentrations and for a short period of time. These responses include an increase in antioxidant enzymes [8], mediated by the Keap1-Nrf2 pathway [9], and the activation of several signaling pathways, such as nuclear factor (NF) κB, mitogen-activated protein kinase (MAPK), and peroxisome proliferator-activated receptor γ co-activator 1α (PGC-1α) [10]. The redox balance is crucial for correct cell function, and maybe an antioxidant supplementation could partially interfere with the oxidative damage caused by exercise promoting the signaling for a better adaptation.

Melatonin (5-methoxy-N-acetyl-triptamine (aMT)) is a ubiquitous molecule synthesized from the amino acid tryptophan. As a hormone, its functions include circadian rhythm regulation (body temperature or sleep–wake cycle), but it appears that its original function may have been to protect cells form the harmful effects of an atmosphere rich in oxygen [11]. Its secretion varies during the lifespan: it is highest during the first to third year and starts declining from adolescence until elderly age, when the levels are the lowest [12]. Exercise induces the secretion of aMT, but only if it is performed during daylight hours, as the luminosity of the daytime is the most important factor regulating its secretion [13,14]. The intensity of exercise is also an important factor. Heavy load exercises (over 75% of the VO_2max_ intensity) induced an immediate increase in plasma aMT concentration [15].

The use of melatonin as a sports supplement has gained scientific support in recent years, particularly because of its ergogenic effects attributed to its antioxidant, anti-inflammatory, and immunomodulatory properties. Melatonin’s capacity to prevent oxidative stress, modulate muscle damage, and mitigate inflammation induced by reactive oxygen and nitrogen species (RONS), as well as its capacity to enhance the gene expression of antioxidant enzymes, makes it a promising supplement for athletes [16]. Recent data suggest its potential as an ergogenic supplement for football players because of its attenuating effect in oxidative stress, inflammation, and muscle damage and due to its lack of potential adverse effects. Nevertheless, the doses used in human studies are low (5–10 mg), which are most commonly used to treat sleep problems. The direct effects of melatonin supplementation on physical performance have not been demonstrated in humans [17].

Research has demonstrated that melatonin reduces biomarkers of muscle damage, such as creatin kinase (CK) and lactate dehydrogenase (LDH); attenuates lipid peroxidation in cell membranes; and downregulates the expression of proinflammatory enzymes, including induced nitric oxide synthase (iNOS), cytokines IL-2, TNF-α, interferon-γ (IFN-γ), and granulocyte-macrophage colony-stimulating factor [16]. It has also been previously described that a single dose of aMT enhances the effects of exercise in cell adaptation through a higher activation of PGC-1a in rats’ muscles, allowing the animals to swim for more time at a anaerobic threshold intensity [18].

In the scenario described above, aMT appears as an endogen molecule that may counteract the effects of oxidative stress induced by a very high-intensity exercise. So, the aim of this study was to evaluate the potential ergogenic and oxidative stress prevention effects of melatonin in a maximal-effort exercise protocol in rats.

## 2. Materials and Methods

### 2.1. Animals

Thirty male *Sprague-Dawley* rats were acquired (Harlan-Ibérica, Barcelona, Spain), with an average weight of 321.3 ± 8.0 g and an average age of 9.5 ± 0.6 weeks. In this work, only male rats were included to avoid any distortion by the ovarian cycle in the response to exercise. Animals were stabled in the Support Service for Animal Experimentation facilities of Universidad de Zaragoza, with 2 rats/cage in a room where ambient temperature was automatically regulated to 22.0 ± 1.0 °C, under a 12:12 h light–darkness cycle. Dark phase started at 8.00 a.m., and light phase at 8.00 p.m., in order to simulate a maximum activity schedule. Water and food were available ad libitum (diet for rodents’ maintenance was provided by RMM, Harlan-Ibérica, Barcelona, Spain). All animal handlings and experimental procedures were performed in the morning in an isolated thermoregulated room, prepared to simulate darkness using red light and covering natural light entrances. Cages were placed inside opaque compartments to avoid exposure to ambient light during transportation. All experiments were carried out in strict compliance with European, national, and regional regulations. The study protocol was approved by the Ethics Committee of Universidad de Zaragoza (ref. PI47/08) and the Clinical Research Ethics Committee of Aragón (ref. CP02/2010).

### 2.2. Experimental Design

The thirty animals were randomly distributed into three groups, homogeneously, with n = 10 rats/group (according to recent similar studies [18,19,20,21,22]): Control group, Exercise group, and Exercise + aMT group. Rats in the Exercise and Exercise + aMT groups carried out a maximum ergometry until exhaustion, in which the maximum speed reached was recorded. To perform these acute exercise tests, a single-lane rat treadmill (Panlab^®^, Cornellà de Llobregat, Barcelona, Spain) was used, with an electrified grid at its rear end and connected to equipment that allowed the speed to be modulated and recorded.

**Figure 1:**
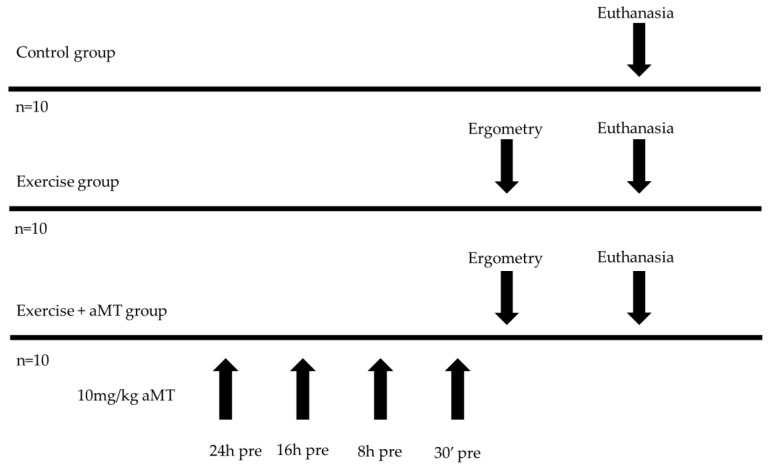
Summary of the experimental design.

For Exercise + aMT group, the administration of melatonin (10 mg/kg weight) was provided according to several authors [18,20,23]. Specifically, aMT was dissolved in <0.1% ethanol and then diluted in NaCl solution (0.9%). Control and Exercise groups received the same volume as vehicle (NaCl 0.9%), and Control group animals also remained at rest. Intraperitoneal administration of both aMT and vehicle was carried out at 24 h, 16 h, 8 h and 30 min before acute exercise test (Figure 1).

Regarding the groups that performed the running test (Exercise and Exercise + aMT), immediately after finishing, each animal was euthanized by CO_2_ exposure before being decapitated. Control group rats were euthanized the next day at the same time slot, following the same procedure. Quadriceps skeletal muscle, brain and liver were immediately extracted and kept at −80 °C until the analytical procedures. This process was performed in less than 10 minutes for each animal.

### 2.3. Exercise Protocol

During the week prior to the ergometric test, Exercise and Exercise + aMT animals carried out an adaptation protocol on the treadmill for 5 days, 1 h/day. In this way, rats ran lightly on the treadmill at a constant speed (0.10–0.12 m/s).

All ergometry tests were performed between 10 a.m. and 12 p.m. The acute exercise test began with a speed of 0.15 m/s and a treadmill inclination of 10°, and speed was increased by 0.03 m/s every 3 min [24]. The maximal effort criterion used to end the ergometry test was defined as the onset of evident fatigue, identified by the rats’ inability to continue running on the treadmill despite the application of electrical stimulation [25]. Initial intensity for electric stimulus was 0.2 mA (the minimum allowed by the ergometric equipment), and the maximum intensity was limited to 0.4 mA at the end of the test.

### 2.4. Analytical Procedures

All the chemicals and solvents, of the highest grade available, were acquired from Sigma (Madrid, Spain). TMA-DPH was obtained from Molecular Probes (Eugene, OR, USA).

To determine the fluidity in each tissue, it was necessary to first proceed with the isolation of all membranes, both cellular and mitochondrial. For skeletal muscle and liver, the procedure previously described by Graham was used [26], with slight modifications in the brain protocol. These methods are based on successive differential centrifugations at different speeds (×*g*) and times (minutes).

To sum up, the different tissues were chopped and placed in a glass homogenizer equipped with a Teflon rotor (Heidolph RZR 2020, Schwabach, Germany). This process was carried out by keeping the samples cold.

For both skeletal muscle and liver tissues, a buffer solution consisting of 0.02 M 4-(2-hydroxyethyl)-piperazine-1-ethanesulfonic acid (HEPES)—0.14 M KCl (pH 7.4) was used. The tissue homogenate was centrifuged at 1000× *g* for 10 min at 4 °C to remove remains of solid tissue and cell nuclei. The supernatant was then centrifuged at 50,000× *g* for 20 min at 4 °C. The pellet, which contained the membranes, was resuspended in HEPES, homogenized and centrifuged again at 10,000× *g* for 10 min at 4 °C. After this centrifugation, the following procedure was carried out: (1) the supernatant and the buffy coat were resuspended and centrifuged at 50,000× *g* for 20 min at 4 °C, to obtain cell membranes; (2) the pellet was resuspended in HEPES and centrifuged at 10,000× *g* for 10 min at 4 °C, to obtain mitochondria membranes. Both final pellets, cell and mitochondrial membranes, were resuspended in 0.05 M tris (hydroxymethyl)aminomethane (TRIS) (pH 7.4). The samples were aliquoted and stored at −80 °C until the assays were carried out.

Regarding brain tissue, this was homogenated at 4 °C in 0.32 M sucrose. This homogenate was centrifuged at 1000× *g* for 10 min at 4 °C to remove remains of solid tissue and cell nuclei. The resulting supernatant was then centrifuged at 30,000× *g* for 20 min at 4 °C. The pellet, containing the membranes, was resuspended in Milli-Q^®^ ultrapure water [27,28], homogenized and centrifuged again at 8000× *g* for 20 min at 4 °C. Next, (1) the supernatant and the buffy coat were resuspended and centrifuged at 48,000× *g* for 20 min at 4 °C, to obtain cell membranes; (2) the pellet was resuspended in Milli-Q^®^ water and centrifuged at 8000× *g* for 20 min at 4 °C, to obtain mitochondria membranes. Both final pellets, cell and mitochondrial membranes, were resuspended in 0.05 M TRIS (pH 7.4). They were distributed in aliquots and stored at −80 °C until further analysis.

Fluidity was monitored from triplicate determinations using TMA-DPH as fluorescent probe. Its incorporation into the plasmatic or mitochondrial membrane and the determination of membrane fluidity were carried out according to that described elsewhere [29]. Tissue cell and mitochondrial membranes (0.5 mg protein/mL) were resuspended in 50 mM TRIS (3 mL final volume) and mixed with TMA-DPH (66.7 nM).

After stirring vigorously on a vortex for 1 min, the preparation was incubated for 30 min at 37 °C. Fluorescence measurements were performed in a Perkin-Elmer LS-55 Luminescence Spectrometer equipped with a circulatory water bath to maintain the temperature at 22 ± 0.1 °C. Excitation and emission wavelengths of 360 and 430 nm were used, respectively. The emission intensity of vertically polarized light was recorded by an analyzer oriented parallel (I_VV_) or perpendicular (I_VH_) to the excitation plane. A correction factor (G) was applied to account for the optical system. Polarization (P) was calculated using the following equation:

$$P=\frac{I_{V_{V}}-{G\;I}_{V_{H}}}{I_{V_{V}}+{G\;I}_{V_{H}}}$$

An inverse relationship exists between membrane fluidity and polarization [29]; thus, membrane fluidity is expressed as 1/P. Protein concentration was determined by the Bradford method using bovine serum albumin as standard [30].

The content of protein carbonyls was measured according to the method of Levine et al. [31]. To the plasma sample, 100 µL of 50 mM TRIS buffer and 200 µL of 10 mM 2,4-dinitrophenylhydrazine (DNPH) solution were added, and the mixture was vortexed, followed by incubation at 37 °C for 1 h. Ice-cold trichloroacetic acid (325 µL) was added to the mixture. The pellet obtained after centrifugation at 3000× *g* for 10 min was washed three times with 1 mL of an ethanol/ethyl acetate mixture (1:1, *v*:*v*). The last pellet was dissolved in 6 M guanidine (700 µL) and incubated again at 37 °C for 15 min. After centrifugation at 12,000× *g* for 10 min, the absorbance of the supernatant was measured spectrophotometrically at 375 nm, and its concentration was expressed as µM carbonyl groups. Guanidine was used as a blank.

MDA + 4-HDA concentrations (µM) were used as an index of the oxidative breakdown of lipids in the plasma [32]. In the assay, MDA + 4-HDA react with N-methyl-2-phenylindole, yielding a stable chromophore with a maximum peak absorbance at 586 nm; 1,1,3,3-Tetramethoxypropane was used as the standard.

### 2.5. Statistical Analysis

Data were analyzed using t-Student’s test to compare means of final speed and repeated measures ANOVA for the other variables. When significant main effects were found, pairwise comparisons were conducted using Tukey’s adjustments for multiple comparisons. Effect sizes were calculated using Hedges’ g and Cohen’s d. Effect size categories were defined as small (d = 0.2), medium (d = 0.5) and large (d ≥ 0.8). Statistical analyses were performed using GraphPad Prism (v9.4.1.681) and SPSS (v29.0). The level of statistical significance was set at *p* < 0.05 for all analyses.

## 3. Results

This section is divided into performance variables and oxidative damage indicators in different tissues according to the aim of the study.

### 3.1. Exercise Performance

**Figure 2:**
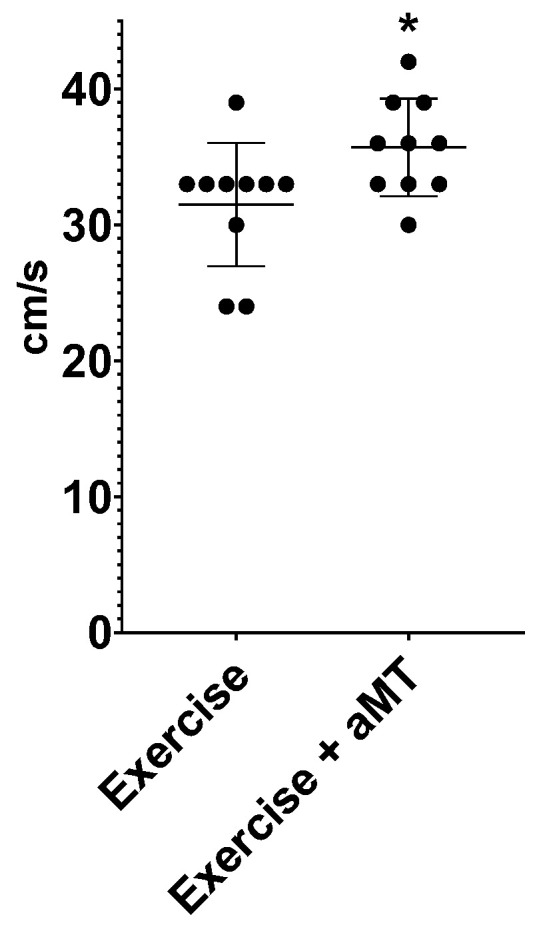
Final speed (cm/s) reached at the end of the incremental ergometry by each animal in group Exercise (without melatonin) and in group Exercise + aMT (animals with melatonin). * means *p* < 0.05.

Our first aim was to evaluate the aMT administration on the performance of the animals during a maximal effort exercise. This potential ergogenic effect is clearly shown in Figure 2, where it can be seen that 6 out of 10 rats treated with melatonin achieved a higher final speed than the median speed of all rats in the Exercise group (33 cm/s). In contrast, only 1 out of 10 rats in the non-melatonin group reached higher speed at the end of the ergometry. When both groups were compared (31 ± 4 vs. 36 ± 4 cm/s), the aMT group reached a significantly higher speed (*p* = 0.0338, Hedges’ g effect size 4.68).

### 3.2. Muscle Damage

To evaluate the oxidate stress prevention effects of melatonin during a maximal effort exercise protocol in rats, markers of lipid and protein oxidative damage and markers of membrane function were analyzed (see Methods section) in three different tissues (muscle, brain and liver).

**Figure 3:**
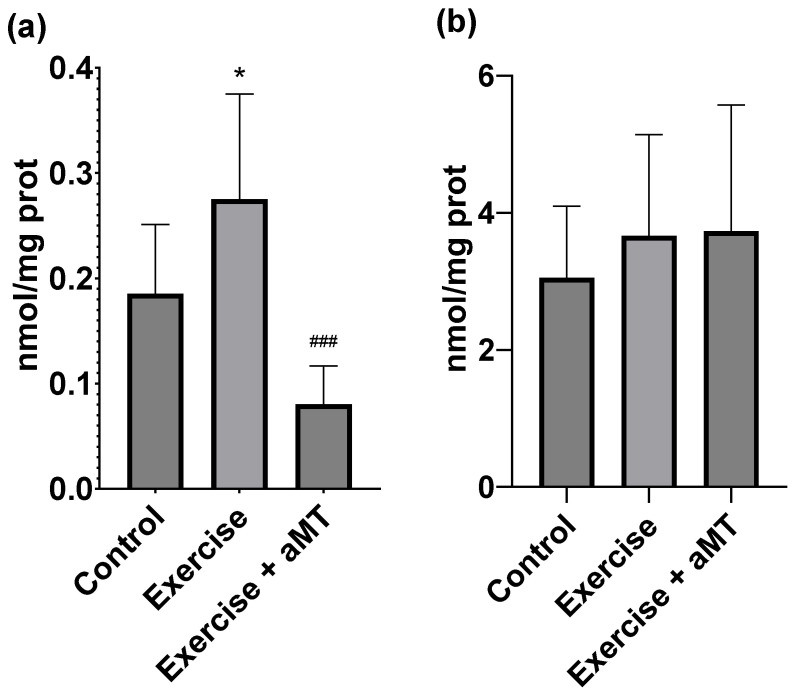

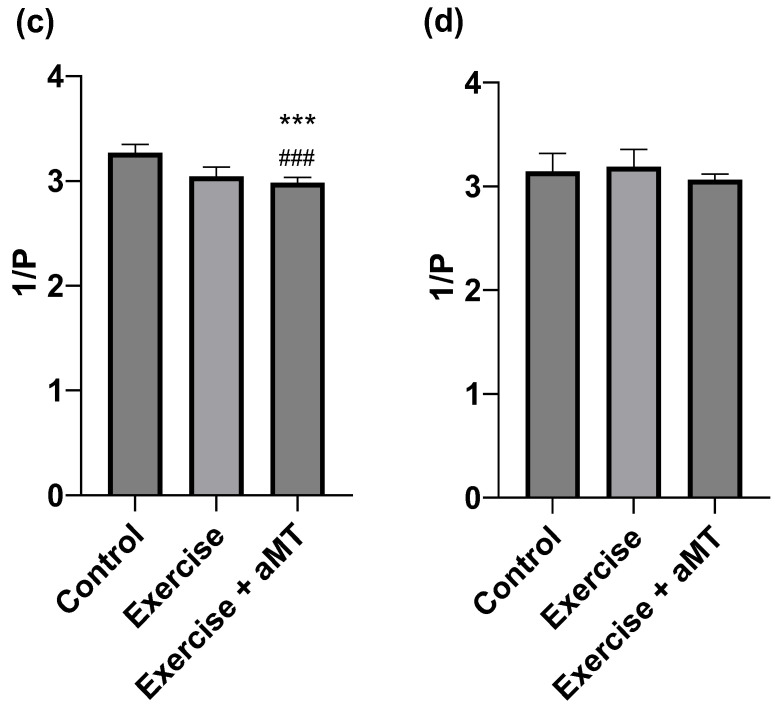
Muscle. (**a**) Malondialdehyde + 4 hydroxyalkenals (nM/mg proteins), (**b**) carbonyl content (nM/mg protein), (**c**) Membrane fluidity (1/P) and (**d**) mitochondrial membrane fluidity (1/P) in group Control (without exercise or melatonin), at the end of the incremental ergometry in group Exercise (without melatonin) and in group Exercise + aMT (animals with melatonin). * means *p* < 0.05 vs. Control. *** means *p* < 0.001 vs. Control. ### means *p* < 0.001 vs. Exercise.

As shown in Figure 3a, MDA concentrations increased in muscle tissue after exercise (0.185 ± 0.065 vs. 0.275 ± 0.099 nmol/mg protein, d = 1.07). However, this effect was prevented when rats were previously administered with aMT (0.275 ± 0.099 vs. 0.080 ± 0.036 nmol/mg protein, d = 2.31). This increase in lipid peroxidation markers was not followed by protein carbonyls. Figure 3b demonstrates that exercise led to a non-significant increase in protein damage, and aMT did not reverse this effect.

Focusing on membrane function, sarcolemma fluidity (Figure 3c) and mitochondrial membrane fluidity (Figure 3d) were analyzed. The effect of aMT on sarcolemma was significant, making it more rigid (2.982 ± 0.051) compared to the Exercise group (3.042 ± 0.090) (d = 0.83) and even to the Control group (3.270 ± 0.078) (d = 3.97). In contrast, mitochondrial membrane fluidity remained unchanged following either exercise or aMT administration.

### 3.3. Brain Damage

**Figure 4:**
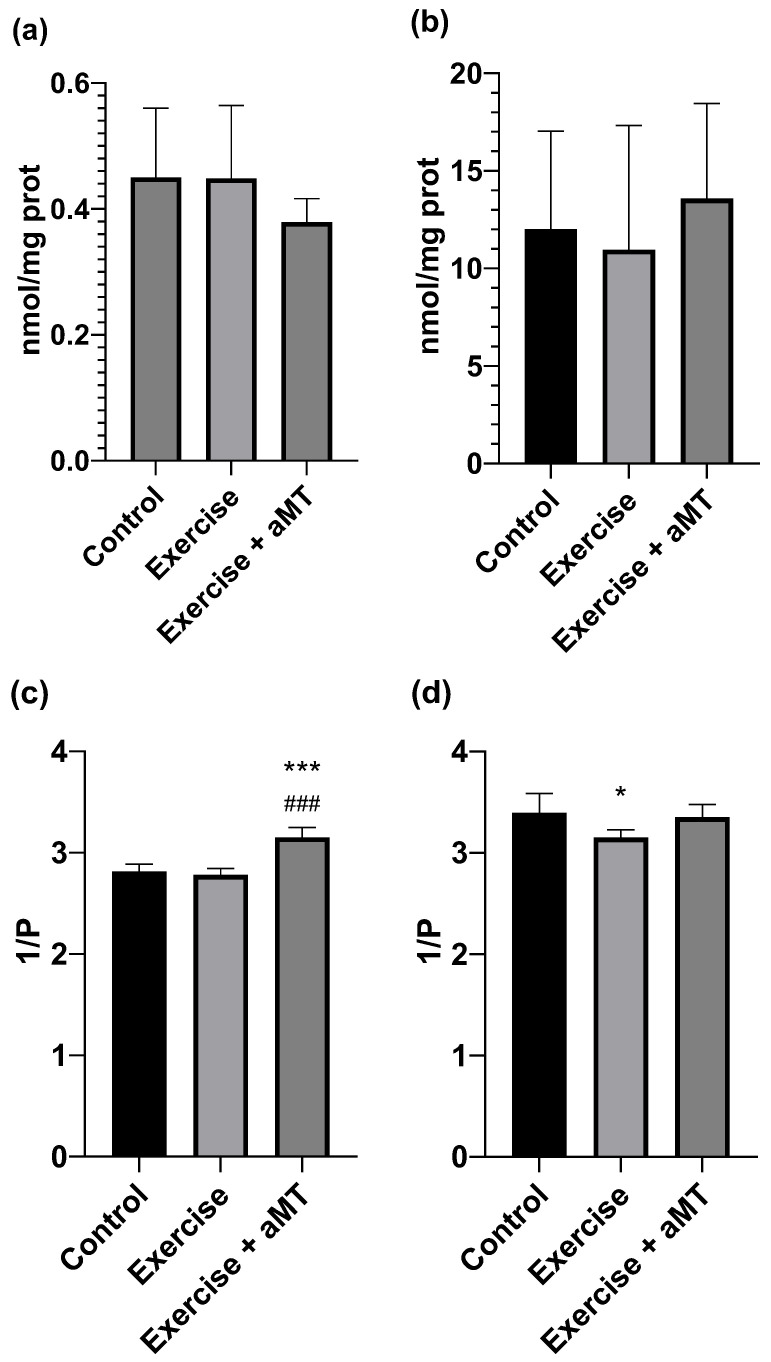
Brain. (**a**) Malondialdehyde + 4 hydroxyalkenals (nM/mg proteins), (**b**) carbonyl content (nM/mg protein), (**c**) membrane fluidity (1/P) and (**d**) mitochondrial membrane fluidity (1/P) in group Control (without exercise or melatonin), at the end of the incremental ergometry in group Exercise (without melatonin) and in group Exercise + aMT (animals with melatonin). * means *p* < 0.05 vs. Control. *** means *p* < 0.001 vs. Control. ### means *p* < 0.001 vs. Exercise.

Analyzing the effects of exercise and aMT administration, no statistically significant effects were observed in MDA and Carbonyl concentrations, as shown in Figure 4a,b. Although a slight decrease was observed in the aMT group compared to the Control and Exercise group, this reduction was not significant.

Regarding membrane fluidity, a significant increase was found in the group treated with melatonin (Control: 2.813 ± 0.074 and Exercise: 2.781 ± 0.061 vs. aMT: 3.151 ± 0.095) (Control vs. aMT d = 4.19), (Exercise vs. aMT d = 4.98) in brain cells membranes (Figure 4c). However, no significant changes were observed in mitochondrial membranes (Figure 4d). Exercise made the mitochondrial membranes more rigid, as expected, while the aMT group showed a slight increase in fluidity, nearly reaching control levels, but this was not significant.

### 3.4. Liver Damage

**Figure 5:**
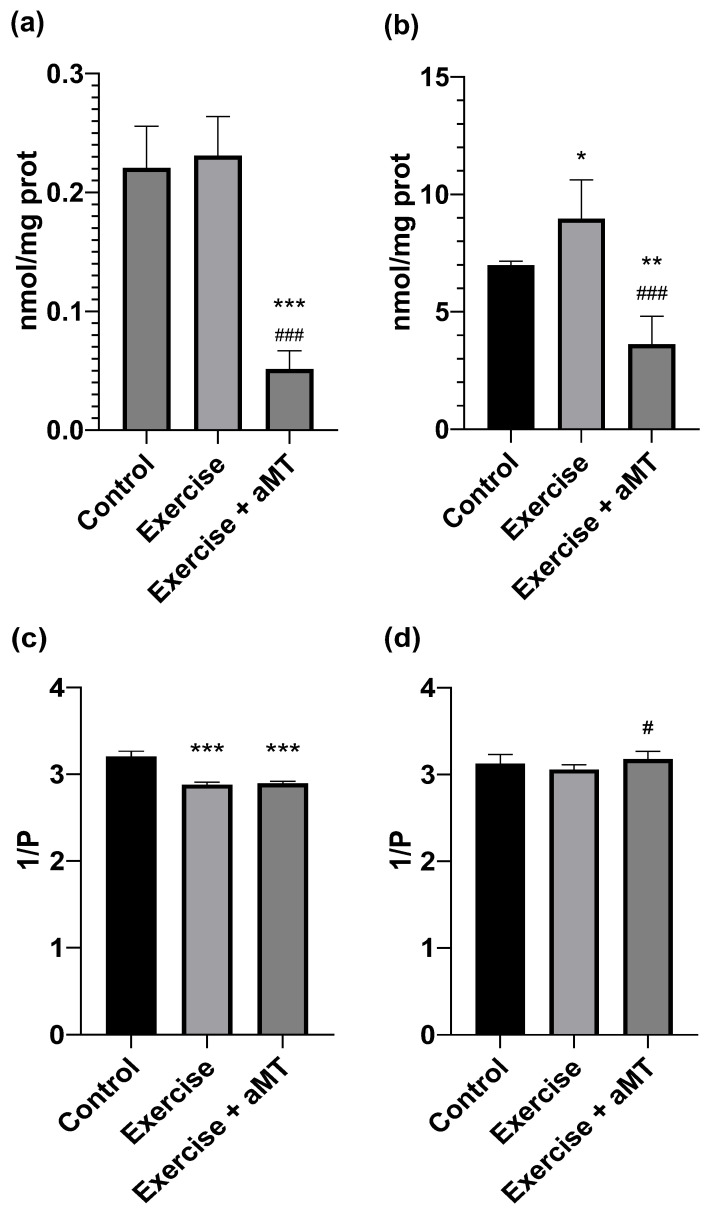
Liver. (**a**) Malondialdehyde + 4 hydroxyalkenals (nM/mg proteins), (**b**) carbonyl content (nM/mg protein), (**c**) membrane fluidity (1/P) and (**d**) mitochondrial membrane fluidity (1/P) in group Control (without exercise or melatonin), at the end of the incremental ergometry in group Exercise (without melatonin) and in group Exercise + aMT (animals with melatonin). * means *p* < 0.05 vs. Control. ** means *p* < 0.01 vs. Control. *** means *p* < 0.001 vs. Control. # means *p* < 0.05 vs. Exercise. ### means *p* < 0.001 vs. Exercise.

As shown in Figure 5a, MDA concentrations slightly and not significantly increased in liver tissue after exercise, and this effect was prevented by the administration of aMT (0.221 ± 0.035 vs. 0.051 ± 0.015 nmol/mg protein) (d = 5.84). The increase in oxidative stress markers was higher in protein carbonyls than in MDA, increasing from 6.980 ± 0.173 to 8.960 ± 1.650 nmol/mg protein after exercise (d = 1.47) (Figure 5b). In the aMT group, protein carbonyl levels were significantly lower (3.615 ± 1.191 nmol/mg protein) (d = 3.55).

Regarding membrane fluidity, exercise induced an increase in the rigidity of liver tissue cell membranes, a change that was not reversed using aMT (Figure 5c). In contrast, a significant increase in mitochondrial membranes was observed in the group treated with melatonin (Figure 5d).

## 4. Discussion

The main findings of the present study are as follows: (1) aMT administration exhibited a clear ergogenic effect in rats performing an exhausting exercise protocol on a treadmill, and (2) this ergogenic effect may be partially attributed to its antioxidant properties.

### 4.1. Running Performance

The most recent review on the influence of melatonin on exercise in humans did not identify any ergogenic effects in the studies analyzed, although the highest dose administered was 8 mg, and 2 × 6 mg [33]. In our study with Sprague-Dawley rats, we selected a much higher dose compared to humans to evaluate the ergogenic potential of melatonin, according to previous research. After reviewing the scientific literature, no evidence of toxicological effects associated with melatonin administration specifically in rats has been reported, even at supraphysiological doses [34]. Based on this, we administrated four doses of 10 mg/kg body weight within 24 h. At these supraphysiological doses, a clear ergogenic effect was observed, as shown in Figure 1, with the rats achieving a higher speed at the end of the selected exercise protocol. Similar results were found where animals perform other types of exercise such as swimming [35]. In that study, different doses and exercise protocols were used, but an increase in performance duration for the exhausting continuous exercise (with no increases in intensity) was described, with no protective effects from tissue damage and/or inflammation. Melatonin administration was also found to be more effective during the wakefulness period in rats. Even though melatonin enhanced performance at any time of day compared to the Control group, the time to exhaustion was longer when rats were more spontaneously active than other periods [36].

### 4.2. Effects on Skeletal Muscle

Studying the adaptive mechanisms induced by physical activity in skeletal muscle tissue is particularly interesting, since muscle contraction is an essential requirement for exercise. After an acute physical effort, MDA + 4-HDA concentration levels were significantly higher in the Exercise group compared to the Control group, indicating a marked increase in oxidation mediated by free radicals and caused by acute exercise. Similar increases in MDA concentrations after an acute exercise protocol in the rat skeletal muscle structure have been reported [37,38], along with increases in thiobarbituric acid reactive substances (TBARS) [39]. Other authors, using various acute physical exercise protocols, have also observed increases in oxidative markers in some muscles, although these changes were not statistically significant [40,41]; some studies reported no changes [42,43]. The most significant finding in the present study occurred in the Exercise + aMT group, since aMT not only reduced muscle lipoperoxidation compared to the Exercise group but also decreased it further, reaching even lower levels of standard lipoperoxidation than those observed in the Control group. This result demonstrates that melatonin is a powerful antioxidant against skeletal muscle lipoperoxidation caused by acute exercise. This effect of aMT is consistent with the results presented in previous studies [44], including those conducted in humans [45,46].

However, regarding the other oxidative stress marker (protein carbonyls), our results do not show significant differences. The mean values of the two groups that performed acute exercise, with and without melatonin, were very slightly higher than those of the Control group. Nevertheless, this increase was not substantial enough to be statistically significant due to the high standard deviation. The literature presents some discrepancies in this regard. While most studies report significant increases in protein carbonylation following high-intensity acute exercise [40], when intensity is moderate-high, significance is missing [47].

Considering the fluidity of the sarcolemma (membrane of muscle fibers), acute exercise induced rigidity in both the Exercise and Exercise + aMT groups in comparison with the Control group, with significant differences observed only in the administered aMT group. TMA-DPH offers important advantages over other fluorescent markers due to its incorporation of a TMA residue, which imparts water solubility to the otherwise hydrophobic DPH molecule. Without the TMA moiety, DPH tends to accumulate in an unstructured manner within the core of the lipid bilayer. In contrast, the amphipathic nature of TMA-DPH allows it to intercalate parallel to the longitudinal axis of phospholipids with its cationic residue oriented towards the surface when added to biological membranes [48]. This property provides a more accurate reflection of the bilayer’s phospholipid dynamics

The increased rigidity in sarcolemma in the Exercise + aMT group could be an inverse consequence of the reduction in muscle tissue lipoperoxidation. As can be seen in Figure 3a, melatonin produced MDA values significantly lower than those of the Exercise group, even decreasing below the values of the Control group, though not significantly. From an antioxidant perspective, this could cause an opposite effect with respect to membrane fluidity, which is mainly composed of lipids. Melatonin, precisely due to its powerful antioxidant action on muscle lipoperoxidation, could modify the lipid composition of cell membranes, which could consequently affect the normal fluidity dynamics of these membranes by overstabilizing the lipids’ structure. Moreover, this apparent rigidity could be due to an increase in the proportion of saturated vs. unsaturated lipids [49,50]. Our results are aligned with those obtained using 5-doxyl-stearic acid (5-DS), suggesting that the rigidity caused by acute exercise mainly affects the outermost part of the lipid bilayer [51]. Similar results occurred in other studies where erythrocyte membranes of different animal species were analyzed, including horses [52], dogs [53] or even rats [54]. Thus, while melatonin demonstrates an antioxidant effect on other markers analyzed, it does not increase fluidity levels in either the sarcolemma or the membrane of muscle fiber mitochondria. Regarding the mitochondria membranes of the muscle fibers, no significant changes were observed. In agreement with our results, another study measured mitochondrial fluidity in four muscles [55], vastus external, gastrocnemius, tibialis and extensor digitorum longus, across three groups of rats that followed strenuous exercise protocols performed on a treadmill. While significant rigidity was noted in the mitochondrial membranes of the vastus externus and gastrocnemius, no significant changes were detected in the tibialis or extensor digitorum longus. These results suggest that mitochondrial fluidity during high-intensity acute exercise could be correlated with the contractile activity degree developed by the muscle group analyzed [37,38,39,40,41,42,43,44,45,46,47].

To summarize the effects on quadriceps skeletal muscle, acute exercise causes significant lipoperoxidation, which is drastically decreased by prior administration of melatonin, even reducing levels below those of the Control group. However, this action of melatonin has not been effective in protein oxidation. Additionally, a marked rigidity of the sarcolemma is observed in this tissue, without melatonin returning the membrane fluidity to its basal values. This rigidity is possibly due to the high demand for the contractile activity of skeletal muscle tissue during physical activity performance, in addition to a possible affectation in the sarcolemma lipid structure due to the melatonin antioxidant action in muscle lipoperoxidation.

### 4.3. Effects on Brain

The brain, as a central nervous system structure, represents an important organ to assess the toxic effects of free radicals, since it is especially vulnerable due to containing high concentrations of polyunsaturated fatty acids, by using an enormous amount of O_2_ to produce ATP, and by its antioxidant enzyme scarcity. Compared with the liver, in the rat brain, the catalase activity is 1/20, while superoxide dismutase (SOD) and glutathione peroxidase (GPx) activity is around 1/3 [56]. In relation to oxidative stress markers both in lipids and proteins, no significant differences were observed in the Exercise group vs. Control, nor the Exercise + aMT group vs. Exercise, so the aMT administration prior to exercise performance did not generate a favorable imbalance in oxidative stress.

Regarding the values of membrane fluidity recorded, melatonin appears to perform a fluidizing function in the membranes of brain tissue, both in cells and in intracellular mitochondria. In cell membranes, aMT increases fluidity not only compared to the Exercise group but also compared to the Control group. Therefore, this suggests that aMT inherently increases the fluidity capacity of brain cells, which could serve as a preventive agent in potential situations of oxidative damage. To date, no studies in the scientific literature have reported on the fluidity of brain cell membranes in rats related to melatonin and exercise. Nevertheless, in a previous study, where the effects of an acute individual test up to exhaustion were examined in various rat central nervous system locations, no significant changes in synaptosomes were found, except in the brainstem [57]. Attending to mitochondria, acute exercise did not produce significant changes either, which could be due to the fact that the central nervous tissue does not develop a great metabolic activity as occurs with other tissues such as skeletal muscle (contractile activity) or liver (purification and catabolism of waste products generated during muscle contraction), while the mitochondria function in nervous tissue is limited to ensuring neurons a constant ATP production, independent of the physical activity level. In our study, melatonin administered before the acute test did not increase the fluidity of mitochondrial membranes compared to the Control group, as it did with the brain cells, but it did cause an increase in the fluidity value compared to the Exercise group (without aMT), being similar to the basal levels of the Control group rats. This protective effect of melatonin on mitochondrial membranes in the brain has been shown in some conditions [58,59], though not specifically in the context of exercise.

### 4.4. Effects on Liver

Based on our results, no statistically significant changes were observed in liver lipoperoxidation in the Exercise group compared to the Control after performing acute physical exercise. In the scientific literature, most experimental models report that acute exercise induced marked hepatic lipid peroxidation [37,60,61,62]. What is remarkable is that, as occurred with the results of LPO in skeletal muscle, the group of animals that performed acute exercise after administering aMT drastically reduced LPO, even well below the standard values of the Control group This antioxidant effect of melatonin on the liver after acute exercise has barely been addressed in the literature, except for some studies with rats performing swimming exercises [63].

Regarding protein carbonylation, the results present a similar trend to those shown for lipoperoxidation, with the exception that the increase in this marker in the Exercise group was statistically significant. While acute exercises taken to the extreme of exhaustion also produced marked carbonylation in rat liver homogenates [62], other studies have not detected hepatic carbonylation, despite following a maximum protocol [60,64]. On the other hand, the aMT antioxidant effect on liver function had already been confirmed [65], but its role as a re-balancer of oxidative stress in proteins from hepatic homogenates after acute exercise has not been demonstrated until now. Its action is very similar to that seen in lipoperoxidation in the same organ, reaching oxidation values in the aMT group statistically lower than the other two groups of animals, the Control and Exercise groups.

In relation to membrane fluidity measurements, as observed in skeletal muscle, a significant rigidity was detected in the two groups that performed acute exercise (Exercise and Exercise + aMT) compared to the Control group. This suggests that, presumably, the lipid and protein molecules comprising the hepatocyte membranes bilayer are affected, regardless of whether melatonin was administered or not. In the scientific literature, the small number of works that assess the effects of physical exercise on the liver cell membranes fluidity is striking. Interestingly, a significant result in our study is the increase in mitochondrial membrane fluidity values in the Exercise + aMT group compared to the Exercise group. This result contrasts with the rigidity observed in cell membranes within the same tissue.

This finding aligns with previous research, where it has been observed that melatonin interacts with the lipid bilayers of the mitochondrial membrane, enhancing the electron transport chain and preventing functional deterioration induced by mitochondrial nitric oxide synthase in rats [66].

On this point, while other elements as antioxidant agents, such as virgin olive oil, sunflower oil [67] or carnitine [68], have been studied, the few studies that have analyzed the exercise effects on liver mitochondrial membranes have also shown increased fluidity. Therefore, the alteration suffered by the cell membranes of hepatocytes, where their fluidity decreases, does not affect the membrane of mitochondrion, as the main organelle involved in the energy and metabolic processes of the cell. In fact, melatonin improves its fluidity.

## 5. Conclusions

Our results suggest that melatonin may have an ergogenic effect in increasing exercise on treadmill until exhaustion, since the group of animals administered aMT achieved a significantly higher final speed compared to the group that performed the ergometry without aMT. This action of melatonin could be due, at least in part, to its potential antioxidant capacity and its water and lipid solubility, resulting in a possible protective agent against damage produced during this type of exercise.

Nevertheless, the antioxidant capacity of melatonin in this protocol has only been partially observed, since its effects on minimizing oxidative stress indicators varied depending on the tissue analyzed. Melatonin was most effective against the following: (1) lipoperoxidation in skeletal muscle and liver; (2) protein carbonylation only in the liver; (3) cellular membrane rigidity in the brain and liver; and (4) mitochondrial membrane rigidity exclusively in the liver, with no significant effects observed in other measurements.These findings highlight the need to expand the range of variables analyzed in each tissue to better evaluate melatonin’s antioxidant capacity under this type of exercise. Additional measurements, such as total antioxidant status (TAS) or the activity of key antioxidant enzymes (e.g., glutathione peroxidase, glutathione reductase, catalase, and superoxide dismutase), could provide further insights. However, it is important to consider the limitation posed by the amount of tissue available in each animal, which restricts the possibility of conducting all potential measurements.

To sum up, the differences between tissues responses to exercise and melatonin administration require further investigation. Future research should also focus on elucidating other possible mechanisms that explain the potential ergogenic effect of aMT administration, as those suggested during the Discussion. Additionally, the limited evidence on the toxicological effects of melatonin may be a point to evaluate the effects of its administration in humans, including the determination of the most appropriate dose and its impact on exercise performance.

## Data Availability

The original contributions presented in the study are included in the article; further inquiries can be directed to the corresponding author/s.
